# Ultrasound-Combined Sterilization Technology: An Effective Sterilization Technique Ensuring the Microbial Safety of Grape Juice and Significantly Improving Its Quality

**DOI:** 10.3390/foods9101512

**Published:** 2020-10-21

**Authors:** Tingting Ma, Jiaqi Wang, Lukai Wang, Yanhao Yang, Wanyi Yang, Haoli Wang, Tian Lan, Qianwen Zhang, Xiangyu Sun

**Affiliations:** 1 College of Food Science and Engineering, College of Enology, Northwest A&F University, Yangling 712100, China; matingting@nwafu.edu.cn (T.M.); hello-wjq@nwafu.edu.cn (J.W.); wlk@nwafu.edu.cn (L.W.); yangyanhao@nwafu.edu.cn (Y.Y.); yangwanyi@nwafu.edu.cn (W.Y.); Wangholy@nwafu.edu.cn (H.W.); lt771451884@nwafu.edu.cn (T.L.); 2 Department of Plant and Soil Sciences, Mississippi State University, Starkville, MS 39762, USA; qz72@msstate.edu

**Keywords:** grape juice, thermosonication sterilization, nisin-assisted thermosonication sterilization, microbial safety, sensory quality, functional indicators

## Abstract

The effects of ultrasound (US), thermosonication (TS), ultrasound combined with nisin (USN), TS combined with nisin (TSN), and conventional thermal sterilization (CTS) treatments on the inactivation of microorganisms in grape juice were evaluated. TS, TSN, and CTS treatments provided the desirable bactericidal and enzyme inactivation, and nisin had a synergistic lethal effect on aerobic bacteria in grape juice while not having any obvious effect on the mold and yeast. Compared with CTS, the sensory characteristics of grape juice treated with TS and TSN are closer to that of fresh juice, its microbial safety is ensured, and the physicochemical properties are basically unchanged. More importantly, the total phenolic content and antioxidant capacity of juice treated with TS and TSN were significantly increased, and the total anthocyanin and flavonoid contents were largely retained. Taken together, these findings suggest that TS and TSN has great potential application value and that it can ensure microbial safety and improve the quality of grape juice.

## 1. Introduction

Grapes are popular among consumers for their beautiful appearance, delicious taste, and high nutritional value, and thus they have been honored as “the queen of fruits” [1]. They is rich in a variety of bioactive substances, such as polyphenols, flavonoids, anthocyanins, plant fiber, and various vitamins and minerals [2], which can protect the skin and cardiovascular and nervous systems as well as providing anti-bacterial and anti-inflammatory effects [3]. Grape juice is one of the most common deep-processing products of grapes, which can reduce albumin and sodium chloride in the kidneys, enhance liver function, promote bile secretion, promote digestion [4], soften blood vessels [5], improve neurasthenia, alleviate excessive fatigue, and prevent heart disease and cancer [6,7]. Hence, in China, grape juice has a reputation as a “plant milk”.

Sterilization is one of the key operations in grape juice processing, and thermal sterilization methods are the most commonly used techniques. At present, conventional thermal sterilization (CTS) is still widely used in the processing of grape juice [8]. Although it can effectively inactivate microorganisms and undesirable enzymes, contributing to a longer shelf life for the fruit juice, high temperature treatments lead to sensory deterioration, a loss of nutrients and functional substances, and a significant reduction in the quality of the fruit juice [9]. These deficiencies, coupled with the increasing consumer awareness about health and nutrition, prompted the food industry to explore new sterilization and preservation technologies. Hence, nonthermal sterilization technologies have become a research focus in the field of food processing in recently years. Ultrasound (US) treatment is one of the innovative nonthermal sterilization technologies that could not only ensure the safety but also improve the quality of liquid food [9]. The effects of US inactivated microorganisms and enzymes are mainly dependent on the mechanical effects (turbulence, liquid circulation, shock waves, high-speed shear force, and micromechanical shocks) and/or chemical effects (formation of free radicals and decomposition of water vapor in the bubbles) of cavitation [10]. The shear-induced cell wall destruction, cell membrane damage, content release, and DNA damage from free radicals generated by thermolytic dissociation leads to overall deactivation [11]. Furthermore, US could also effectively retain beneficial nutrients and bioactive compounds, especially heat-sensitive components, while decreasing the microbial load and endogenous enzyme activity and minimally affecting the physiochemical and sensory indexes in liquid foods [12]. However, using US alone still had some disadvantages, such as high energy consumption, which was mainly due to the high-power, long-term over-processing of US treatment [13], etc., which limited the application of US in juice processing.

Meanwhile, coupling the US with other sterilization techniques showed good application potential in the preservation of various juices [11,14,15,16,17]. A variety of US-combined sterilization technologies not only show synergistic effects in inactivating microorganisms and enzymes, reducing the high energy cost of US, but also maintained the nutritional, organoleptic, and physicochemical properties of juices, even better than US treatment alone. Currently, the US-combined sterilization technologies that have been reported are thermosonication (TS, that is, US plus mild heat) [18,19], manosonication [20], manothermosonication [15], US plus gassing [16], US plus irradiation [17], US plus pulsed electric field [21], and US/TS plus antibacterial agents [22], etc. TS and US/TS plus an antibacterial agent (such as nisin) have been widely addressed [18], mainly due to their simple operation and low cost. Mild heat treatment employed a lower heating temperature during heat processing, which could reduce or even eliminate the degradation of heat-sensitive nutrients and bioactive components, hence preventing the juice quality deterioration caused by high heating temperatures [23]. Nisin is a peptide composed of 34 amino acid residues that is produced by strains of *Lactococcus lactis* subsp *lactis*, which has a wide range of activities against Gram-negative and Gram-positive bacteria, and it is widely used in the storage and preservation of liquid food [24]. It is one of the biological bacteriostatic safety agents recognized and recommended by the World Health Organization for food preservation and been commercialized in the form of dry concentrated powder [25]. Therefore, US combined with nisin (USN) and TS combined with nisin (TSN) might, in principle, complement one another’s disadvantages and focus their advantages to obtain high-quality juices with good sterilization treatment.

So far, there are still few studies on the effects of ultrasound-combined sterilization technology on grape juice. Thus, the aim of this study was to comparatively investigate the impacts of ultrasound-related treatments and CTS on the overall quality of grape juice to (i) compare the bactericidal effects of US, USN, TS, TSN, and CTS, and (ii) to evaluate the effects of TS, TSN, and CTS on the inactivation of enzymes, physicochemical properties, functional characteristics, and sensory quality of grape juice. Additionally, the relationships between the antioxidant capacity of grape juice and the color properties and functional substances were also determined. This study could provide some technical support and a theoretical basis for the production of high-quality grape juice.

## 2. Materials and Methods

### 2.1. Chemicals and Reagents

All the standards and reagents, including 2-octanol, gallic acid, 1,1-diphenyl-2-picrylhydrazyl (DPPH), and 6-hydroxy-2,5,7,8-tetramethylchroman-2-carboxylic acid (Trolox), were purchased from Sigma-Aldrich (St. Louis, MO, USA). All medium, including the plate counting medium, lauryl sulfate peptone broth, bright green lactose bile broth, and Bengal red medium, were purchased from Hope Bio-Technology Co., Ltd. (Qingdao, China).

### 2.2. Grape Juice Preparation

Fresh grapes (No. 8 Hutai, a characteristic grape variety in Shaanxi, China) were purchased from a local market in Yangling, China. These grapes were washed and pressed mechanically using a juice extractor (H-AE-DNBI19, Seoul Hurom Co. Ltd., Seoul, Korea). The squeezed juice was centrifuged at 4 °C, 3000× *g* for 5 min, and the supernatant was used as fresh grape juice, then poured into 250 mL of sterilized glass bottles, sealed with sterile membranes, and stored at 4 °C until further treatment.

### 2.3. Nisin Solution Preparation

In accordance with the method by Liao et al. [11] with minor changes, 0.5 g of Nisin Z with a potency ≥900 IU/mg was dissolved in 100 mL of pasteurized grape juice, filtered through a 0.22 μm filter to remove any microorganisms, and stored at 4 °C for later use.

### 2.4. Sterilization Process

The fresh grape juice was subjected to CTS, US, USN, TS, or TSN treatment, and fresh grape juice was used as a blank control group (CK). The single treatment volume of the juice was 50 mL, and each treatment was used in at least 3 parallel experiments. For CTS treatment, the grape juice samples were sterilized at 85 °C for 10 min [26]. The ultrasound-related treatments were based on the method by Liao et al. [11] with minor changes. The US treatments were conducted using an ultrasonic processor with a built-in probe (ATPIO-1000D, Nanjing Xianou Ltd., Jiangsu, China). The maximum power input was 1000 W, the frequency ranged from 20 to 25 kHz, and the diameter of the ultrasonic horn was 6 mm. The ultrasound probe was submerged to a depth of 15 mm. The applied power level was adjusted to 70% of the maximum power, with a pulse duration of 2 s, on and 3 s off. During the TS treatment, the container was connected to a water bath with a thermostat (XODC-0515-II, Nanjing Xianou) to help it reach up to and stabilize at the indicated temperature of 55 °C, and the other conditions are the same as those in the US treatment. For the USN and TSN treatment, nisin solution was first added to grape juice to reach a final concentration of 200 ppm, and then the same operations as those used for US and TS were performed, respectively. After each treatment, 70% (*v*/*v*) alcohol and sterile water spray were used successively to wipe the ultrasonic probe and the double-walled cylindrical container to avoid cross-contamination.

### 2.5. Microbiological Assay

The determinations of the total bacterial count (TBC), *Escherichia coli*, mold, and yeast were performed in accordance with Chinese national standards GB 4789.2-2016, GB 4789.3-2016, and GB 4789.3-2016, respectively. The results are expressed as the log CFU/mL and log MPN/mL.

### 2.6. Enzyme Activity Assay

Enzyme extract was prepared by dissolving 4% (*w*/*v*) polyvinyl pyrrolidone (PVPP), 1% (*v*/*v*) Triton x-100 and 1 M NaCl in 0.2 M phosphate buffer (pH = 6.5). Grape juice and enzyme extracts (1:1, *w*/*w*) were mixed, extracted at 4 °C for 2 h, and centrifuged at 8952 g/min at 4 °C for 30 min. The supernatant was used to determine the activities of the polyphenol oxidase (PPO) and peroxidase (POD) [27], and the enzyme activity measurement was performed according to Marszałek et al. [28] with slight modifications.

### 2.7. Physicochemical Indicators

The total soluble solids (TSS) were determined as °Bx using a PAL-1 digital Abbe Refractometer (ATAGO Co., Tokyo, Japan). The pH values were measured using a PHS-3E pH meter (Shanghai Leici Co. Ltd., Shanghai, China). The browning indexes (BI) were determined by UV-1780 spectrophotometer (Shimadzu, Kyoto, Japan). The viscosity was measured by NDJ-5S rotary viscometer (Shanghai Pingxuan Co. Ltd., Shanghai, China).

### 2.8. Functional Indicators

#### 2.8.1. Determination of Total Polyphenol Content (TPC), Total Anthocyanin Content (TAC), and Total Flavonoid Content (TFC)

The TPC, TAC, and TFC were determined by Folin–Ciocalteu colorimetric method [29], pH differential method [30], and aluminum chloride colorimetric assay [21,31] results are expressed as µg gallic acid equivalents (GAE)/mL (µg GAE/mL), mg cyanoside-3-glycoside (CGE)/L (mg CGE/L), and mg catechin equivalents/L (mg CTE/L), respectively.

#### 2.8.2. Antioxidant Capacity Assay

The antioxidant capacity of the grape juice samples was determined by DPPH method and Ferric ion reducing antioxidant power (FRAP) method based on previously reports [29], results are expressed as μM Trolox/mL.

### 2.9. Organoleptic Properties

#### 2.9.1. Color Measurement

**Figure 1:**
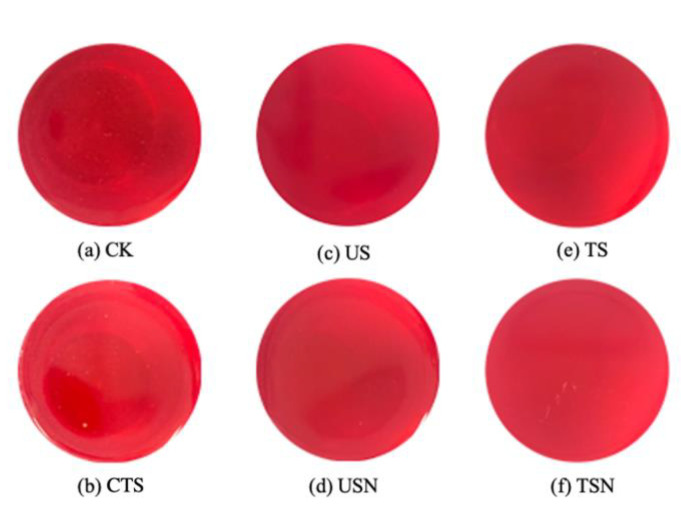
Pictures of grape juice after different sterilization treatments. (**a**) control group (CK), (**b**) conventional thermal sterilization (CTS), (**c**) ultrasound (US), (**d**) ultrasound combined with nisin (USN), (**e**) thermosonication (TS), (**f**) TS combined with nisin (TSN).

The color characteristics of the grape juice samples were determined with a Ci7600 colorimeter (X-rite, Grand Rapids, MI, USA) in transmission mode [11] using CIELab technology. The illuminant is D65 (Artificial Daylight 6500K) provided by a pulsed xenon lamp, which had been calibrated. The pictures after treatments were shown as Figure 1.

#### 2.9.2. Sensory Evaluation

The sensory evaluation was based on the method of Khandpur et al. [10] with slight modification with 24 professionals (12 males and 12 females, from 20 to 25 years old) from Northwest A&F University who had received sensory assessment training participated in the sensory assessment by evaluated for 7 different attributes, namely, appearance, color, smell, astringency, acidity, sweetness, and overall acceptability.

#### 2.9.3. Electronic Nose (E-Nose) Assay

The E-nose assay was conducted using a PEN 3 E-nose (Airsense Analytics, Schwerin, Germany) containing ten metal oxide semiconductors [32]. The specific parameters of the E-nose detection were as follows: the carrier gas velocity was 300 mL/min, the detection time duration was 60 s, and the cleaning time was 240 s with at least 5 times test.

#### 2.9.4. HS-SPME-GC-MS Assay

The volatile components in the grape juice samples were analyzed by headspace solid-phase microextraction (HS-SPME) combined with gas chromatography-mass spectrometry (GC-MS) based on previous studies [33]. The volatile compounds were extracted from the headspace to the SPME fiber (50/30 μm, DVB/CAR/PDMS, Supelco, Bellefonte, PA, USA) for 30 min. The SPME fiber was then injected into the injection port of the GC-MS system and desorbed at 250 °C for 2 min. The volatile compounds were identified and quantitatively analyzed by GC-MS-QP 2010 (Shimadzu, Kyoto, Japan). The National Institute of Standards and Technology (NIST) 14 library database was used to match the retention time and degree of the volatile compounds, and the substances with matching degrees higher than 85% were selected as the effective aroma components. The volatile compounds were then preliminarily identified. The linear retention index (RI) that was calculated from the mixture of n-alkanes (C_8_–C_20_) was used to confirm the identification results. The contents of volatile components in the grape juice samples with different sterilization treatments were quantitatively identified by internal standard method (2-octanol).

### 2.10. Statistical Analysis

Data analysis was performed using SPSS 23, RStudio-1.1.463, Excel 2016, and Origin 9.1. The experimental results are expressed as the means ± standard deviations (SD) of three replicates for each treatment. Multigroup comparisons of the means were carried out by one-way analysis of variance (ANOVA) test with post hoc contrasts by least significant difference (LSD) test and Duncan test. The statistical significance for all tests was set at *p* < 0.05.

## 3. Results and Discussion

### 3.1. Lethal Effects of Different Sterilization Treatments on the Microorganisms

Grape juice is rich in nutrition, and it easily breeds spoilage and pathogenic microorganisms. The TBC, *Escherichia coli*, yeast, and mold were selected as microbial detection indicators in grape juice after different sterilization treatments.

#### 3.1.1. TBC and *Escherichia coli* Counts

**Figure 2:**
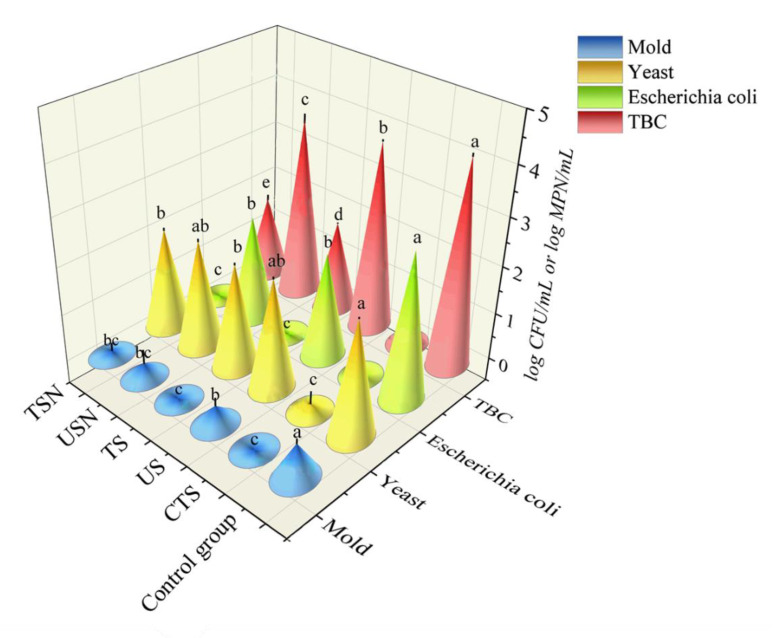
Lethal effects of different sterilization treatments on the microorganisms in grape juice. The *Escherichia coli* results were expressed as log MPN/mL, and the other results were expressed as log CFU/mL.

The TBC and *Escherichia coli* in CK were at 4.25 log CFU/mL and 3.04 log MPN/mL, respectively. The TBC and *Escherichia coli* were reduced to 0, 3.89, 1.87, 3.73, and 1.70 log CFU/mL and 0, 2.18, 0, 2.18, and 0 log MPN/mL after CTS, US, TS, USN, and TSN treatments, respectively. These findings indicated that CTS treatment was still the most effective sterilization method and had the most thoroughly lethal effect on the microorganisms. The TBC and *Escherichia coli* fatality rates of US, TS, USN, and TSN were 56.58%, 99.59%, 69.94%, 99.72%, and 86.36%, 100%, 86.36%, and 100%, respectively. This indicates that US treatment alone showed limited bactericidal effects on TBC and *Escherichia coli*. However, the US-combined treatment (USN and TSN) significantly enhanced the bactericidal effect, which was consistent with Liao et al. [11] and Ma et al. [33]. CTS treatment relies on a thermal effect to destroy bacterial proteins, nucleic acids, and enzyme systems for sterilization purposes [34]. The US depends on the acoustic cavitation effect, which causes the breakage of cell walls as well as damaging the DNA, ultimately leading to the deactivation of different microorganisms [10]. In addition, the effect of nisin on the target bacteria is exerted at the cytoplasmic membrane. Nisin forms pores that disrupt the proton motive force and the pH equilibrium, causing ion leakage and ATP hydrolysis and resulting in cell death [35]. Meanwhile, Figure 2 show that nisin has no synergistic lethal effect on *Escherichia coli*, and Liao et al. [11] also observed that nisin has almost no inhibitory effect on Gram-negative bacteria.

According to Chinese national standard GB7101-2015, when the TBC and the *Escherichia coli* concentration is less than 2 log CFU/mL and 1 MPN/mL, respectively, the juice is considered safe to drink. Hence, CTS, TS, and TSN treatments can ensure the microbial safety of grape juice in terms of TBC and *Escherichia coli* (Figure 2).

#### 3.1.2. Yeast and Mold Counts

The initial yeast and mold counts in CK were 2.40 and 0.73 log CFU/mL, respectively (Figure 2). After the CTS, US, TS, USN, and TSN treatments, the yeast and mold were reduced to 0.12, 2.31, 2.18, 2.26, and 2.11 log CFU/mL and 0, 0.43, 0, 0.30, and 0 log CFU/mL, respectively. Similarly, CTS had the best lethal effect on yeast and mold. The amount of mold in grape juice was always within the microbial safety range. Nevertheless, compared with CK, mold were significantly reduced after TS and TSN treated (*p* < 0.05). Furthermore, there was no significant difference between TS, TSN treatment and CTS treatment on the lethal effect on mold (*p* > 0.05). Compared with CK, yeasts was significantly reduced after treated by TS and TSN (*p* < 0.05). However, the effect of the TS and TSN was significantly lower than CTS (*p* < 0.05). In addition, adding nisin led to a weaker synergistic lethal effect on yeast and mold, similar to Liao et al. [11].

In all, CTS, TS, and TSN treatments showed good sterilization effects, which can effectively guarantee the microbial safety of grape juice. Hence, these three treatments were selected for further research.

### 3.2. Passivation Effects of Different Sterilization Treatments on the Enzyme Activity

**Figure 3:**
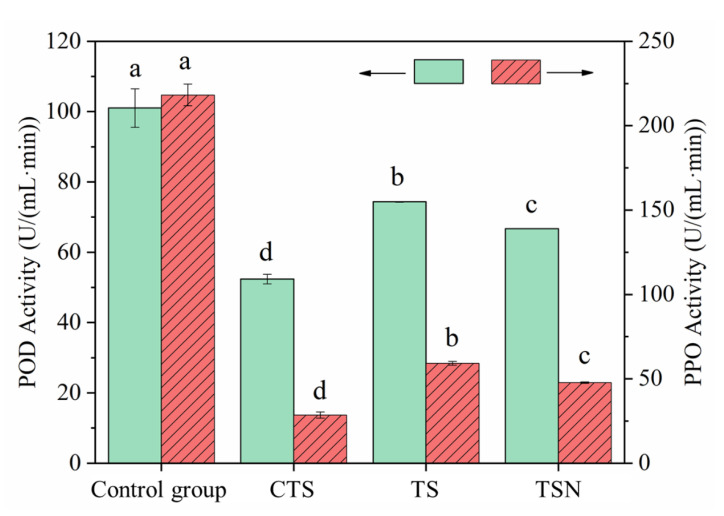
Passivation effects of different sterilization treatments on the enzyme activity of grape juice.

The inactivation of POD and PPO is necessary for improving the color retention and shelf life of fruit and vegetable juices [27]. As shown in Figure 3, the POD22 and PPO activities of CK were 101.02 and 218.17 U/(mL·min), respectively. After CTS, TS, and TSN treatment, these activities were reduced to 52.38, 74.36, 66.70, and 28.51, 59.21, and 47.70 U/(mL·min), respectively. This suggested that CTS still showed the strongest enzyme inactivation effect, while the two US-combined sterilization treatments (TS and TSN) also showed satisfactory enzyme inactivation effects. In addition, it was also found that the combined TS and nisin (TSN) treatment could significantly enhance the enzyme passivation effect. However, the mechanism of synergistic enzyme passivation by nisin still requires further study.

The enzymes in fruit juice are mostly macromolecular proteins. Ultrasound mainly destroys the molecular structure through powerful shock waves or jets [10]. When combined with Nisin, Nisin can form pores in the cell membrane, destroying the power of protons and pH balance to make an impact [24,35]. The effects of ultrasonic treatment on important nutrients mainly include (i) the cell walls and vacuoles of plant tissues are destroyed or colloidal particles around cavitation collapse [10,14] and capillary effect [36], so that soluble nutrients are released; (ii) destroying the molecular structure of nutrients [10,11]. In the whole process, the former is much greater than the latter so that nutritionally important compounds can be retained to the maximum or even increased [14,15,16,17,37].

In general, the CTS, TS, and TSN treatments can effectively inactivate the POD and PPO activity in grape juice and improve the color retention and shelf life of grape juice, and TSN treatment is significantly better than TS treatment (*p* < 0.05).

### 3.3. Effects of Different Sterilization Treatments on the Physicochemical Properties

#### 3.3.1. TSS and pH Analysis

**Figure 4:**
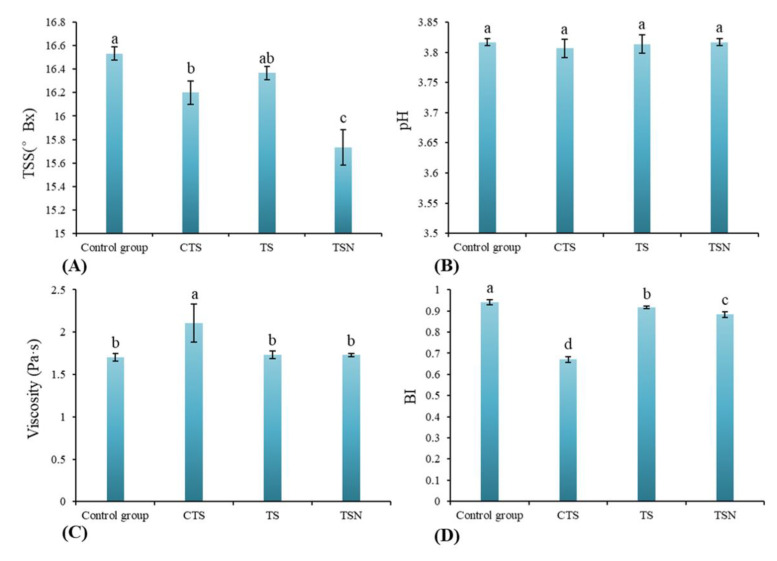
Effects of different sterilization treatments on the physicochemical properties of grape juice. (**A**) Total soluble solids (TSS); (**B**) pH; (**C**) browning indexes (BI); and (**D**) viscosity.

The TSS and pH are two important indicators that affect the sweetness and acidity of grape juice. After CTS and TSN treatment, TSS significantly decreased (*p* < 0.05) in comparison with CK, while there was no significant difference between the TS and CK (*p* > 0.05) (Figure 4A). Wang et al. also draw a similar conclusion in kiwi juice [37] and strawberry juice [38]. The initial pH value of grape juice in CK was 3.82 (Figure 4B). In general, the pH of the juices after all different sterilization treatments showed a relatively stable trend (*p* > 0.05), which is consistent with [39].

#### 3.3.2. BI

As shown in Figure 4C, the BI decreased significantly after all different sterilization treatments; that is, the sterilization treatment reduced the browning degree of the grape juice. The decrease in the BI after CTS was the most significant, followed by TSN. Many studies have also demonstrated that sterilization treatment can inhibit the browning of juices, thereby maintaining or even reducing the BI [11,14,40,41]. One possible reason is that juice browning can be divided into two types, enzymatic browning and nonenzymatic browning. Sterilization treatments may aggravate nonenzymatic browning in juice. Additionally, the sterilization process also effectively passivates various enzymes related to enzymatic browning in the juice, thus significantly reducing the enzymatic browning of the juice, and with the combined effect, the BI decreased.

#### 3.3.3. Viscosity

There were no significant differences in the viscosity of the grape juice samples among the TS and TSN group and CK (*p* > 0.05) (Figure 4D), which indicated that TS and TSN treatment did not change the apparent viscosity of grape juice. However, the viscosity in CTS group increased significantly (*p* < 0.05), which may be due to the complex polymerization of macromolecules caused by the thermal effect, thus affecting the apparent viscosity [42].

### 3.4. Effects of Different Sterilization Treatments on the Functional Properties

#### 3.4.1. TPC

**Figure 5:**
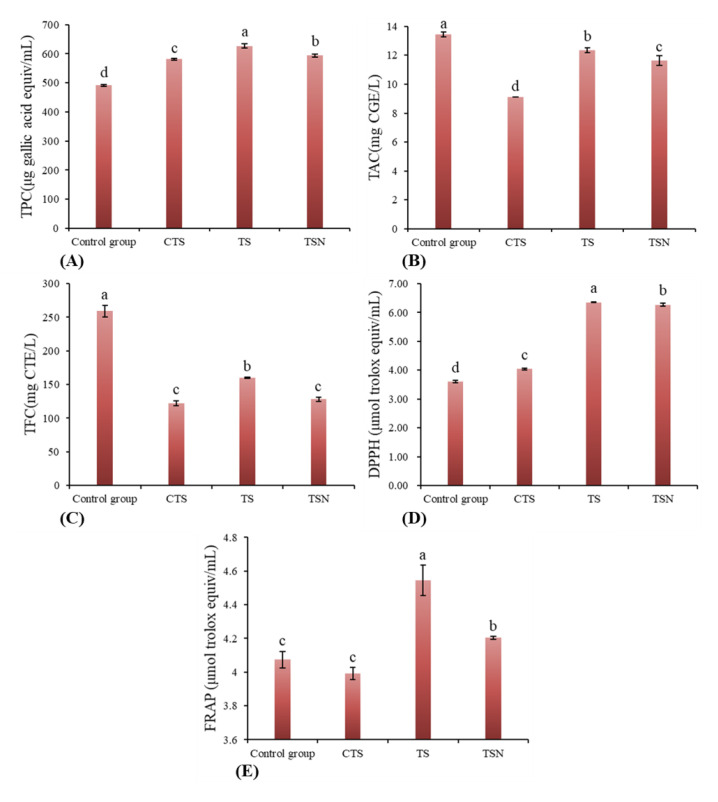
Effects of different sterilization treatments on the functional indicators of grape juice. (**A**) Total polyphenol content (TPC); (**B**) Total Anthocyanin Content (TAC); (**C**) Total Flavonoid Content (TFC); (**D**) DPPH scavenging activity; and (**E**) FRAP assays.

As shown in Figure 5A, the TPC was enhanced significantly after all different sterilization treatments (*p* < 0.05), among which TS showed the most significant effect, which increased TPC from 491.63 to 626.96 μg GAE/mL (27.45%), followed by TSN (20.48%). Similar observations of the enhanced TPC were also reported in US-treated kiwifruit juice [37], strawberry juice [38], and rosehip nectar [39]. This result occurred principally because the US or US-combined treatment could lead to the disruption of cell walls and vacuoles of plant tissues and lead to a cavitational collapse in the surrounding colloidal particles, which all contribute to the dissolution and release of phenolic compounds into the juice [37]. Additionally, a high-intensity US treatment might produce nascent hydroxyl groups, which could be combined with the aromatic rings to produce more phenolic compounds [21].

#### 3.4.2. TAC and TFC

TAC (Figure 5B) and TFC (Figure 5C) suffered varying degrees of loss after all sterilization treatments. The decreases in the TAC or TFC during the sterilization process have previously been reported in CTS-treated pomegranate juice [43], US-treated apple juice [40], and US-treated grape juice [44]. Nevertheless, in comparison with CTS, considerably higher retention rates of TAC (91.73%) and TFC (61.75%) can be observed after TS, followed by TSN with TAC and TFC for 86.47% and 49.51%, respectively. Considering these three functional indexes, TS or TSN treatment could retain the functional components of grape juice to the maximum extent, and thus is a novel sterilization method with high nutritional retention characteristics.

#### 3.4.3. Antioxidant Activity

The DPPH and FRAP assays are the most commonly used methods for determining antioxidant capacity in the food industry. The DPPH value of CK was 3.61 μM Trolox/mL, and in comparison with CK, DPPH value with all different sterilization treatments increased significantly (Figure 5D). The TS-treated showed the highest DPPH ability (6.35 μmol μM Trolox/mL), and they were 76.03% greater than that CK. Followed by TSN group (6.27 μM Trolox/mL) and CTS group (4.05 μM Trolox/mL), which increased by 73.68% and 12.05%, respectively. In addition, there was no significant differences in FRAP between CTS-treated juice samples and CK (*p* > 0.05) (Figure 5E), while TS and TSN significantly improve FRAP value. These results indicate that the US-combined treatment (TS, TSN) not only significantly increased the DPPH radical scavenging activity, but it also enhanced the ferric ion-reducing antioxidant power, which was mainly due to the release and increase of antioxidants (e.g., polyphenols) and the inactivation of some oxidases after US treatment [45].

### 3.5. Correlation Analysis of Antioxidant Activity

**Figure 6:**
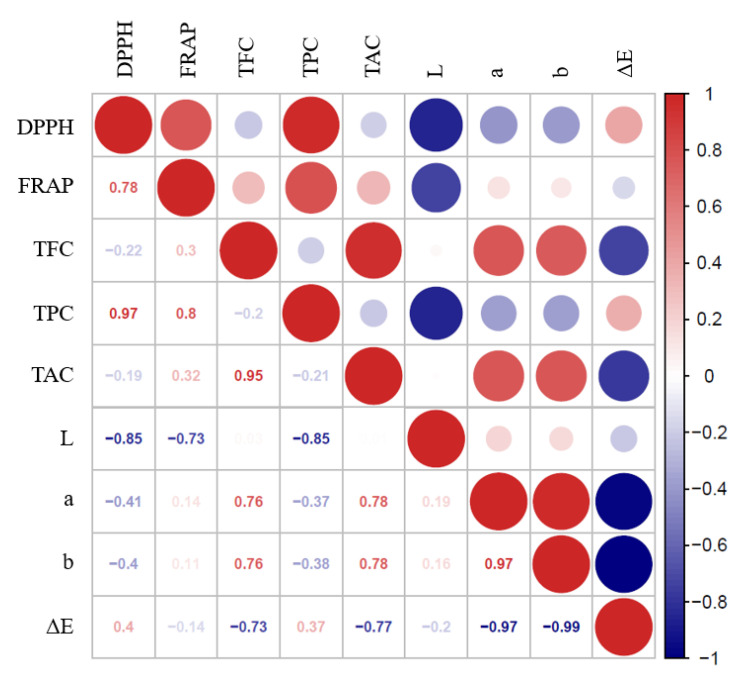
Correlation between the color attributes, antioxidants, and antioxidant capacity of grape juice.

Several studies have reported that polyphenols and flavonoids might contribute to the antioxidant activity of fruit juice, and the color attributes might also be related to the antioxidant activity [38]. The correlation between the color attributes, antioxidants, and antioxidant capacity of grape juice was displayed in Figure 6. There was a significant negative correlation between the L* value and antioxidant activity, and no significant correlation among antioxidant capacity and other color attributes (a*, b*, and ΔE). This suggested that color attributes are not the main factor that contributes to the antioxidant capacity. It has been reported that the antioxidant capacity of fruit juice is mainly related to the antioxidants in the juice samples [37]. In this study, a positive significant correlation was observed between the TPC (R_DPPH_ = 0.97, R_FRAP_ = 0.80) and the antioxidant capacity, while no significant correlation was observed between the TFC (R_DPPH_ = −0.22, R_FRAP_ = 0.30), TAC (R_DPPH_ = −0.19, R_FRAP_ = 0.32) and the antioxidant capacity. Hence, the enhanced antioxidant capacity of grape juice might be attributed to the increase in polyphenols, which indicated that TPC played a prominent role in increasing the antioxidant capacity. Similar findings had also been reported in kiwifruit juice [37] and strawberry juice [38].

### 3.6. Effects of Different Sterilization Treatments on the Sensory Quality

#### 3.6.1. Color Analysis

The color properties are also important indicators used to evaluate the juice quality. A pleasant color can also improve consumer acceptance. Compared with CK, the L* value of grape juice after TS and TSN treatment decreased significantly (*p* < 0.05) (Table 1), which was similar with US-treated orange juice sample [10]. In addition, the a* and b* values decreased significantly after all different sterilization treatments, which indicated that sterilization treatment led to significant green/blue changes in juice color, among which CTS had the most significant impact while TSN had the least. Currently, there is still controversy about the effects of different sterilization treatments on the a* and b* values of juice. Khandpur et al. [10] reported that a* and b* values in US-treated orange juice clearly decrease. Conversely, Liao et al. [11] and Cruz-Cansino et al. [19] found a* and b* values in TSN-treated apple juice and TS-treated cactus pear juice tended to increase. Therefore, further research is still needed.

In addition, US treatment can also cause cell disruption, resulting in the release of intracellular compounds, which might affect the final color of grape juice [38]. A noticeable difference could be observed between two colors when the ΔE value is greater than 2. CTS-treated showed a most obvious color changes compared to CK (22.51), which significantly affects the sensory performance, while TS (14.53) and TSN (11.24) showed much lower color changes.

#### 3.6.2. Artificial Sensory Evaluation

**Figure 7:**
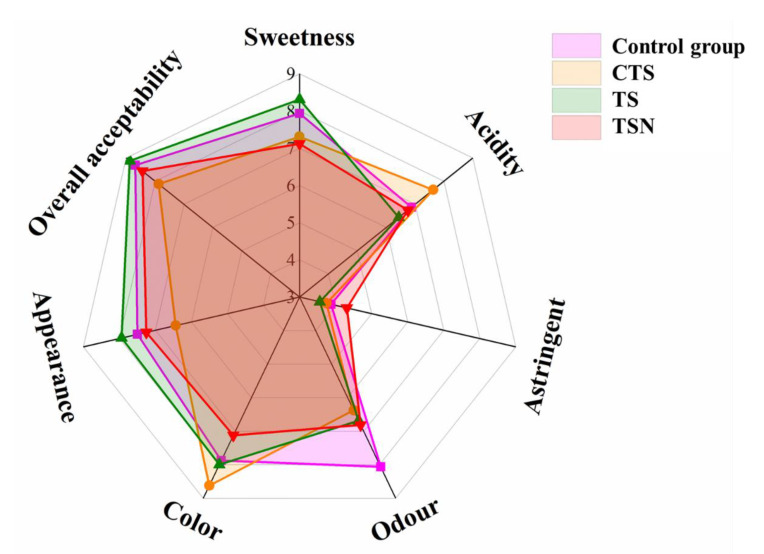
Spider plot of artificial sensory evaluation on grape juice under different sterilization treatments.

The sensory characteristics have an important impact on foods acceptance by consumers. As Figure 7 shows, the overall acceptability of grape juice in the TS group was the highest, which was due to its more suitable acidity and sweetness, lowest astringency, and color. Further, its appearance was closest to that of CK. In addition, CTS had the lowest overall acceptability, which was mainly due to the significantly negative effect of the CTS treatment on the appearance and color and the relatively obvious sour taste. In addition, the odor characteristics changed significantly after all different sterilization treatments, and the odor of TSN was closer to CK, while the odor change in CTS was the most obvious. Apichartsrangkoon et al. [46] and Wang et al. [47] also found that the sterilization process, especially the CTS, could significantly change the odor characteristics of the juice, thus causing changes in the juice quality.

#### 3.6.3. E-Nose Analysis

**Figure 8:**
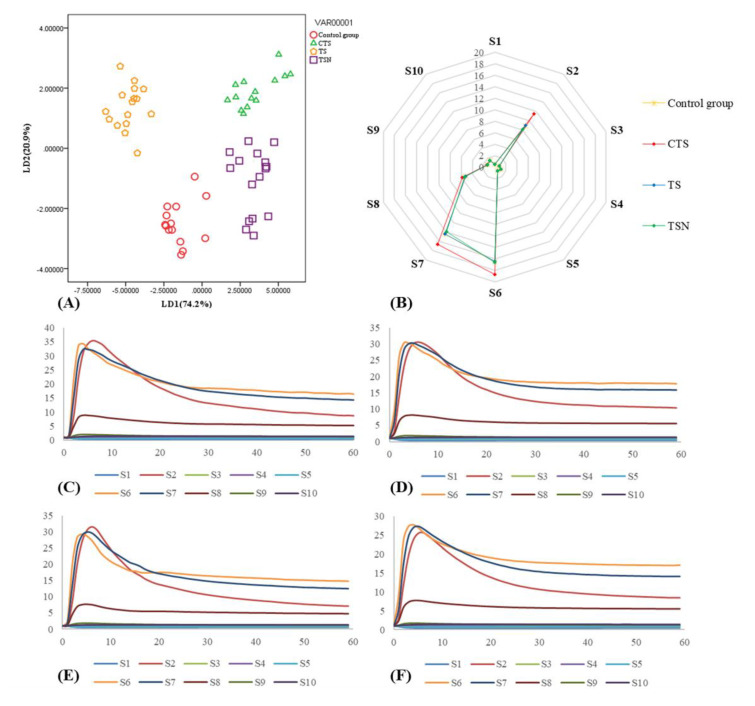
E-nose assay of grape juice after different sterilization treatments. (**A**) Linear discriminant analysis (LDA) results of grape juice under different sterilization treatments; (**B**) radar chart of E-nose response data of grape juice after different sterilization treatments; (**C**) sensor responses recorded using ten sensors in the E-nose for fresh grape juice; (**D**) sensor responses recorded using ten sensors of the E-nose for conventional thermal sterilization (CTS); (**E**) sensor responses recorded using ten sensors of the E-nose for TS; and (**F**) sensor responses recorded using ten sensors of the E-nose for TSN.

The sensor response was recorded using ten sensors of the E-nose for CK (Figure 8C), CTS (Figure 8D), TS (Figure 8E), and TSN (Figure 8F). The average stable signal of each sensor at 56–60 s of an E-nose was used for linear discriminant analysis (LDA) analysis [48], results are shown in Figure 8A.

The two discriminant functions explained 95.1% of the total variance, including 74.2% by LD1 and 20.9% by LD2. Overall, the E-nose with LDA models could distinguish among grape juice samples with different sterilization treatments. The E-nose response data on grape juice after TSN treatment was the closest to that of CK, which indicated that the odor characteristics of the TSN group were the closest to those of CK. This result is consistent with the results of the artificial sensory evaluation (Figure 7). In addition, the distribution of E-nose response data from CTS was significantly different from CK, indicating that the odor characteristics of CTS-treated grape juice changed significantly, which may be due to the increase or formation of some nitrogen oxides (S2), methyl compounds (S6), and inorganic sulfide (S7) in CTS-treated grape juice (Figure 8).

#### 3.6.4. GC-MS Analysis

Based on the results of the artificial sensory evaluation (Figure 7) and E-nose (Figure 8), the odor characteristics of grape juice under different sterilization treatments changed significantly. To explore the impacts of the sterilization treatments on the volatile components in grape juice further, GC-MS was used to analyze and compare the changes in the volatile components. The results are presented in Table 2.

A total of 36 volatile compounds were identified in 4 different groups of grape juices, with 7 species of alcohols, 2 species of terpenes, 8 species of esters, 5 species of aldehydes, 2 species of ketones, and 2 phenols as well as other ethers, hydrocarbons, and acids. In general, the volatile compounds and their concentrations were significantly different in different sterilization groups. A total of 26 volatile compounds were identified in CK, of which 3-methyl-4-oxopentanoic acid was the main component, accounting for 61.57% of the total volatile compounds, followed by aldehydes, accounting for 20.01% of the total volatile compounds; the dominant aldehydes were hexanal (5.70%) and (E)-2-hexenal (14.31%). In addition, the types and contents of esters and alcohols also affected the aroma characteristics of grape juice to a certain extent. Table 2 shows that the main alcohols and esters in grape juice are ethanol, isopropyl alcohol, and hexenol and its isomers, including 1-hexanol as well as isopropyl acetate, butanoic acid, ethyl ester, (E)-2-butenoic acid, ethyl ester and hexanoic acid, ethyl ester, etc. This profile is similar to that in previous studies [49,50]. However, the terpene and hydrocarbon compounds in grape juice identified in this study are inconsistent with those reported in the literature [49,50], which might be due to the different grape varieties, origin, ripeness, and detection method.

As shown in Table 2, 31, 28, and 31 volatile components were identified in the CTS, TS, and TSN groups, respectively. On the whole, the main components of the terpenes, alcohols, and esters (such as ethanol, 3-methyl-4-oxopentanoic acid, hexanal, (E)-2-hexenal, 1-hexanol, hexanoic acid, and ethyl ester) in CK were significantly higher than those in other three groups, which indicated that the sterilization methods in this experiment significantly reduced the contents of the main volatile components in the grape juice. Similar conclusions were also observed in Filho et al. [51]. In addition to the reduction in the contents of the main volatile components, sterilization treatment also resulted in the formation of some new substances. For example, the CTS group produced acetone, 2-hexenal, and 1-methyl-4-(methylethylidene) cyclohexene, while the TSN group produced isopropyl acetate. Additionally, (Z)-3-hexen-1-ol and (E)-2-hexen-1-ol could not be detected in the CTS group after sterilization treatment. The research of Filho et al. [51] and Fan et al. [52] also proved that the alcohols and aldehydes in juices would be degraded and isomerized during sterilization. According to the E-nose results, some nitrogen oxides (S2), methyl compounds (S6), and inorganic sulfides (S7) in the CTS-treated grape juice were increased or formed. However, no consistent rules were observed in the detection of GC-MS; therefore, these three sensors in the E-nose might also be sensitive to and respond to other substances besides these three kinds of substances, which are included in this study. In general, no significant regular changes were observed in each monomer of volatile substance in grape juice after the different sterilization treatments, and the effect of sterilization on the aroma characteristics of grape juice may be due to the complex changes in the overall volatile composition of grape juice. In the future, further studies are still needed, such as using GC-MS-O and sensomics to seek these changed volatile components with low contents but high thresholds, and using HPLC or HPLC-MS to seek these changed nonvolatile components. These components might respond to the S2, S6, and S7 sensor of E-nose, to illuminate the mechanism through which sterilization caused changes in the aroma characteristics of grape juice.

## 4. Conclusions

The research conducted a comprehensive study on the differences in the quality attributes (including microbiological assay, enzyme activity, physicochemical indicators, functional indicators, antioxidant capacity, and organoleptic properties) of grape juice under different sterilization treatments. The research has found on the basis of ensuring the microbial safety of grape juice, the US-combined sterilization technology could not only maintain the sensory quality and physicochemical parameters of juice products, but it also significantly improved the nutritional and functional characteristics of the juice products. The research also conducted a preliminary exploration on the impact of ultrasonic treatment on the content of biologically active substances and clarified the correlation between the antioxidant capacity of grape juice and its color attributes and various functional substances. Therefore, US-combined sterilization technology can be considered as a potential novel nonthermal technique. It could help the food industry to produce high-quality juice, and it holds good promise for commercial applications.

## Figures and Tables

**Table 1:** Color parameters changes of grape juice after different sterilization treatments.

Processing Method	Color			
	L*	a*	b*	ΔE
Control group	56.67 ± 2.32 ^a^	15.05 ± 0.30 ^a^	26.31 ± 0.23 ^a^	0.00 ± 0.00 ^d^
CTS	56.13 ± 0.70 ^a^	7.50 ± 0.52 ^c^	5.12 ± 0.85 ^d^	22.51 ± 0.99 ^a^
TS	48.30 ± 0.62 ^c^	10.60 ± 0.18 ^b^	15.32 ± 0.59 ^c^	14.53 ± 0.06 ^b^
TSN	51.42 ± 0.43 ^b^	11.09 ± 0.11 ^b^	17.19 ± 0.97 ^b^	11.24 ± 0.95 ^c^

Note: values with different letters in the same column are significantly different (*p* < 0.05) from each other.

**Table 2:** The variety and concentration of the volatile compounds in grape juice under different sterilization treatments.

NO	Ret. Time	Concentration (µg/L)					CAS	RI
		Compounds	Control Group	CTS	TS	TSN		
1	4.293	(S)-l-Alanine ethylamide	2.65 ± 0.096 a	1.91 ± 0.062 c	2.27 ± 0.043 b	2.69 ± 0.066 a	71773-95-0	1097
2	4.517	Acetaldehyde	1.27 ± 0.087 bc	1.64 ± 0.080 a	1.22 ± 0.091 c	1.40 ± 0.020 b	75-07-0	408
3	4.79	Ethanol	105.52 ± 7.113 a	89.10 ± 5.007 b	91.46 ± 5.047 b	98.94 ± 2.419 ab	64-17-5	463
4	4.997	Acetone	-	2.26 ± 0.090 a	-	-	67-64-1	455
5	5.063	Isopropyl Alcohol	5.67 ± 0.372 a	4.95 ± 0.100 ab	4.53 ± 0.708 b	4.92 ± 0.426 ab	67-63-0	482
6	5.343	Acetic acid, methyl ester	-	-	1.93 ± 0.025 a	2.06 ± 0.100 a	79-20-9	487
7	6.52	3-methyl-4-oxo-Pentanoic acid	733.41 ± 23.161 a	654.13 ± 11.903 b	615.57 ± 6.538 c	672.90 ± 11.100 b	6628-79-1	1046
8	7.57	Isopropyl acetate	6.29 ± 0.780 a	5.77 ± 0.361 ab	4.71 ± 0.181 c	5.39 ± 0.180 bc	108-21-4	621
9	8.637	Triethylamine	-	-	-	2.36 ± 0.265 a	121-44-8	667
10	8.98	n-Propyl acetate	3.55 ± 0.108 a	2.98 ± 0.035 b	2.73 ± 0.119 c	2.85 ± 0.040 bc	109-60-4	686
11	10.64	Propanoic acid 2-methyl-ethyl ester	1.42 ± 0.036 a	1.13 ± 0.010 b	0.96 ± 0.015 c	0.98 ± 0.026 c	97-62-1	721
12	11.89	Hexanal	67.91 ± 0.392 a	64.81 ± 0.934 b	50.46 ± 0.123 d	54.36 ± 0.672 c	66-25-1	806
13	12.193	Butanoic acid ethyl ester	24.14 ± 1.125 a	20.99 ± 0.066 b	16.63 ± 0.203 d	18.51 ± 0.050 c	105-54-4	785
14	13.683	2-Hexenal	-	1.96 ± 0.061 a	-	-	505-57-7	814
15	13.84	(E)-2-Butenoic acid ethyl ester	12.17 ± 0.686 a	10.11 ± 0.290 b	8.42 ± 0.271 c	8.15 ± 0.015 c	623-70-1	793
16	13.997	(E)-2-Hexenal	170.46 ± 8.243 a	148.82 ± 1.460 b	114.75 ± 0.051 d	132.93 ± 2.033 c	6728-26-3	814
17	14.397	Butanoic acid 2-methyl-ethyl ester	2.06 ± 0.162 a	1.70 ± 0.017 b	1.34 ± 0.111 c	1.48 ± 0.010 c	7452-79-1	820
18	14.517	3-Hexen-1-ol	-	1.91 ± 0.107 b	1.96 ± 0.030 b	2.09 ± 0.067 a	544-12-7	868
19	14.523	(Z)-3-Hexen-1-ol	2.22 ± 0.127 a	-	1.97 ± 0.032 b	1.98 ± 0.015 b	928-96-1	868
20	14.997	(E)-2-Hexen-1-ol	3.64 ± 0.136 a	-	2.36 ± 0.059 b	-	928-95-0	868
21	15.003	(Z)-2-Hexen-1-ol	-	2.49 ± 0.083 b	-	2.69 ± 0.044 a	928-94-9	868
22	15.14	1-Hexanol	9.25 ± 0.127 a	6.48 ± 0.134 c	-	8.58 ± 0.096 b	111-27-3	860
23	16.853	methoxy-phenyl-Oxime	4.83 ± 0.396 bc	5.10 ± 0.256 ab	4.56 ± 0.030 c	5.40 ± 0.021 a	0-00-0	1301
24	20.427	2-Octanone	2.58 ± 0.015 a	2.39 ± 0.026 b	2.44 ± 0.087 b	2.18 ± 0.042 c	111-13-7	952
25	20.947	Hexanoic acid ethyl ester	10.0 ± 0.059 a	9.23 ± 0.222 b	5.95 ± 0.100 d	6.48 ± 0.177 c	123-66-0	984
26	21.65	2,2-dimethyl-Decane	-	3.21 ± 0.272 a	2.71 ± 0.021 b	1.63 ± 0.036 c	17302-37-3	1130
27	22.28	1-methyl-4-(methylethylidene)-cyclohexene	-	1.35 ± 0.010 a	-	-	586-62-9	1052
28	22.4	o-Cymene	2.26 ± 0.256 b	2.98 ± 0.135 a	2.06 ± 0.095 b	2.04 ± 0.025 b	527-84-4	1042
29	22.833	D-Limonene	7.28 ± 0.095 a	7.92 ± 0.081 a	7.82 ± 0.749 a	7.42 ± 0.252 a	5989-27-5	1018
30	23.08	Bis(2-chloro-1-methylethyl) ether	3.22 ± 0.079 a	2.66 ± 0.082 b	2.09 ± 0.015 c	2.20 ± 0.165 c	108-60-1	1016
31	23.193	1,1’-oxybis 3-chloro-Propane	1.15 ± 0.006 a	0.94 ± 0.015 b	0.77 ± 0.117 c	0.92 ± 0.032 b	629-36-7	1144
32	24.013	gamma-Terpinene	1.20 ± 0.049 b	1.68 ± 0.031 a	0.82 ± 0.046 c	0.75 ± 0.076 c	99-85-4	998
33	24.433	3,7-dimethyl-Decane	0.57 ± 0.006 a	0.55 ± 0.072 ab	0.48 ± 0.032 b	0.56 ± 0.010 a	17312-54-8	1086
34	29.1	Decanal	-	1.51 ± 0.378 a	-	0.97 ± 0.087 b	112-31-2	1204
35	37.703	3,5-bis(1,1-dimethylethyl)-Phenol	6.54 ± 0.411 b	8.89 ± 0.092 a	-	-	1138-52-9	1555
36	37.71	2,4-Di-tert-butylphenol	-	-	9.29 ± 0.240 a	7.33 ± 0.270 b	96-76-4	1555

Note: The compounds were identified by comparison of retention indices and the mass spectrum with those of NIST library. Retention indices relative to C_8_-C_20_ n-alkanes on the DB-1MS column. Volatiles are shown according to their order of appearance in the chromatogram on the DB-1MS column—represents not detected. Values with different letters in the same column are significantly different (*p* < 0.05) from each other.
